# Morocco's policy choices to achieve Universal health coverage

**DOI:** 10.11604/pamj.2015.21.53.6727

**Published:** 2015-05-25

**Authors:** Khalid Tinasti

**Affiliations:** 1 Global Health Programme, Graduate Institute of International and Development Studies, Geneva, Switzerland

**Keywords:** Universal health coverage, RAMED, AMO, health governance, prepayment

## Abstract

Morocco's health system remains weak in spite of the improvement of other development indicators in the last ten years. Health remains one of the major challenges to lower the social disparities that are the priority for the authorities. Despite the goodwill of all stakeholders, significant reforms implemented respond only partially to the needs of the population. Morocco established several public insurance schemes, of which one focuses on the poorest, to achieve financial-risk protection for its population. Nevertheless, achieving universal health coverage through one of its dimensions is not sufficient, and all the effort being concentrated in one area has shown the deterioration of equity in access to and quality of health services. Moreover, the insurance schemes did not reach their objectives of protecting a majority of Moroccans from financial hardship.

## Commentary

In the midst of the Arab Spring in 2011, Morocco adopted a new Constitution addressing health in seven different articles, among which article 31 states the right to universal access to health services and the right to financial-risk protection and article 154 stating the right to access quality health services. These Constitutional articles refer directly to the three objectives of universal health coverage (UHC) as promoted by the World Health Organization to achieve universal access to effective health services (including prevention, promotion, treatment and rehabilitation) people need without exposing them to financial hardship [1]. The Moroccan health system suffers a large number of deficiencies. These include no more than 6.5% of the GDP spent on total health expenditures, 24% of the population facing difficulties in accessing health services, inadequate health sector governance [2], and an acute shortage of healthcare workers (6.2 physicians per 10.000 people) [2]. The National Initiative on Human Development, a hypothetically bold and integrated government plan to eradicate poverty supervised directly by the Royal Cabinet does not include or even mention health despite addressing most of its social determinants. Among the 22 countries and territories of the East-Mediterranean region (WHO EMRO), Morocco ranks among the five lowest-ranking on the adult health and health expenditure component of the Human Development Index. Moreover, out-of-pocket health expenditure reached 88.3% (percentage of private expenditure on health) [3] between 2009 and 2013. Populations that have no other option than out-of-pocket payments to access health facilities suffer financial hardship and sometimes avoid resorting to health services because they cannot afford paying for them [1]. Facing several shortcomings in the three dimensions of UHC, along with economic and social realities, Moroccan authorities decided to set priorities through the “progressive realization” [4] of UHC by focusing on financial-risk protection and launching new mechanisms to cover health expenditures with prepayment schemes to avoid direct payments [5]. The health system reform of 2002 focused solely on the financial coverage of health expenses, rather than including equitable access to quality health services.

Law 65-00 of 2002 on health insurance established two prepaid funds systems, the Mandatory Medical Insurance (AMO) and the Medical Assistance Scheme (RAMED) [5]. The former was designed to protect the labour force from financial risk, while the latter focused on the most vulnerable parts of the population to avoid financial catastrophe. AMO, launched in 2005, is a social insurance scheme covering public and private sector employees. It builds on the two former pooling mechanisms: the National Social Security Fund (CNSS) for the private sector and the National Fund for Social Welfare Organisms (CNOPS) for civil servants and employees of the public sector [6]. AMO covered more than 7.6 million people, 4.7 million people in the private sector and 2.9 million people in the public sector by the end of 2012 [6]. This national insurance scheme pools its funds directly from the wages of the beneficiaries’ of which contributions increased by 10% between 2006 and 2012 [6]. RAMED is the national coverage system that protects the most vulnerable populations from health-related out-of-pocket expenses. Eligibility is based on household revenues. Under the scheme the poorest are exonerated from any payment for a large set of interventions: from vaccination, reproductive, maternal, newborn and child health, dental and reconstructive surgeries, access to medications and treatments, to access to emergency rooms [5]. To be eligible the revenues of the household should not exceed 5650 MAD (675 US$) annually [7]. RAMED was launched in January 2013 after a pilot experiment in the rural region TadlaAzilal that started in 2008. It is expected to cover up to 8.5 million, or 28% of the Moroccan population, of which 4.5 million people are considered vulnerable with annual revenues between 3767 MAD and 5650 MAD (450 to 675 US$) and those in absolute poverty earning less than 3767 MAD (450 US$) annually. The former contribute to the scheme with 120 MAD (15 US$) per household annually while the latter receive the services for free.

The scheme is funded up to 75% from the State budget and the remaining is from the contributions of the beneficiaries’ vulnerable category and the local municipalities’ budgets [5–7]. In order to reach a “third quarter” of the population the government launched another mandatory scheme called INAYA in 2007 for the self-employed categories of the population and other craftsmen. The optional nature of this scheme has not attracted the targeted population and in 2009 only 700,000 craftsmen were participants of this scheme [8]. Other populations, such as students, simply do not appear in the strategic choices of policy-makers. Assuming that RAMED achieves its objective of covering the 8.5 million poorest people and that its funding by the State is effectively sustained, including the beneficiaries of AMO, the population covered with financial-risk protection would not exceed 50% of the 33 million Moroccan citizens [3], meaning that half of the Moroccans are left paying directly from their pockets for health services or not using them to avoid financial catastrophe. At the global level 150 million people experience financial catastrophe and 100 million people suffer impoverishment every year because of out-of-pocket health expenses [1]. Enormous efforts have been made to cover the financial-risk of the poorest populations, but the compulsory prepaid schemes are not delivering the expected results [6]. Focusing all the national effort on one dimension of UHC proved insufficient and the need to have an integrated strategy became obvious. The two pathways of “progressive universalism” towards UHC, as identified by Jamison and Summers et al., need to build on the financial protection of the poor, but also on the quality and the availability of a sufficient set of interventions [9]. One of the major challenges in the Moroccan health sector that remains unaddressed is governance. The Ministry of Health is the unique decision-making body which allocates discretionarily resources. The regional health agencies depend on the centralized decisions made in the capital city of Rabat. Communities do not take part in programme implementation, and worse, health is one of the most corrupted sectors in Morocco [2]. Equity in health is under-documented when it comes to sub-Saharan immigrants. In a traditional society, women suffer from their social status, distance from primary care clinics in rural areas, and from violence inside or outside their households [2]. Moreover, the quality of medical care is one of the biggest shortages in the Moroccan health system [10], be it in primary care clinics, prescription and use of medicines, or in the continuity of care.

The Moroccan government faces arduous economic challenges, with a fiscal deficit that reached 6% and a central government debt of 59.6% of the GDP in 2012 [3]. Nevertheless, the government decided to raise the specific excise tax it imposes on tobacco and alcohol in 2014. It is not however specified that the new pooled revenues will benefit the health system directly or be used to reduce the fiscal deficit. Such innovative funding mechanisms are an efficient tool to raise revenues for health, and taxes on harmful products such as tobacco, alcohol and gas are an option [1] that proved effective elsewhere. In Morocco's efforts to tackle its social problems, UHC, if fully implemented, can be an efficient tool to combat poverty and disparities. UHC reduces the burden of diseases on the most vulnerable parts of the population, protects them from impoverishment and therefore lowers the levels of social inequalities. It is the responsibility of Morocco's policymakers to demonstrate their willingness to achieve UHC in all of its dimensions by making policy choices that target the shortages in the current health system and, most importantly, by having a much needed comprehensive and multi-sectoral approach to health.
